# Alteration of Wnt5a expression and of the non-canonical Wnt/PCP and Wnt/PKC-Ca^2+^ pathways in human osteoarthritis osteoblasts

**DOI:** 10.1371/journal.pone.0180711

**Published:** 2017-08-04

**Authors:** Xavier Martineau, Élie Abed, Johanne Martel-Pelletier, Jean-Pierre Pelletier, Daniel Lajeunesse

**Affiliations:** Unité de recherche en Arthrose, Centre de recherche du Centre hospitalier de l’Université de Montréal (CRCHUM), Montréal, Québec, Canada; National Cancer Center, JAPAN

## Abstract

**Objective:**

Clinical and *in vitro* studies suggest that subchondral bone sclerosis due to abnormal osteoblasts (Ob) is involved in the progression and/or onset of osteoarthritis (OA). Human Ob isolated from sclerotic subchondral OA bone tissue show an altered phenotype, a decreased canonical Wnt/β-catenin signaling pathway (cWnt), and a reduced mineralization *in vitro*. In addition to the cWnt pathway, at least two non-canonical signaling pathways, the Wnt/PKC and Wnt/PCP pathway have been described. However, there are no reports of either pathway in OA Ob. Here, we studied the two non-canonical pathways in OA Ob and if they influence their phenotype.

**Methods:**

Human primary subchondral Ob were isolated from the subchondral bone plate of tibial plateaus of OA patients undergoing total knee arthroplasty, or of normal individuals at autopsy. The expression of genes involved in non-canonical Wnt signaling was evaluated by qRT-PCR and their protein production by Western blot analysis. Alkaline phosphatase activity and osteocalcin secretion (OC) were determined with substrate hydrolysis and EIA, respectively. Mineralization levels were evaluated with Alizarin Red Staining, Wnt/PKC and Wnt/PCP pathways by target gene expression and their respective activity using the NFAT and AP-1 luciferase reporter assays.

**Results:**

OA Ob showed an altered phenotype as illustrated by an increased alkaline phosphatase activity and osteocalcin release compared to normal Ob. The expression of the non-canonical Wnt5a ligand was increased in OA Ob compared to normal. Whereas, the expression of LGR5 was significantly increased in OA Ob compared to normal Ob, the expression of LGR4 was similar. Wnt5a directly stimulated the expression and production of LGR5, contrasting, Wnt5a did not stimulate the expression of LGR4. Wnt5a also stimulated the phosphorylation of both JNK and PKC, as well as the activity of both NFAT and AP-1 transcription factors. The inhibition of Wnt5a expression partially corrects the abnormal mineralization, OC secretion and ALPase activity of OA Ob.

**Conclusion:**

These data indicate that the alteration of Wnt5a, a non-canonical Wnt signaling activator, is implicated in the modified signalisation and phenotype observed in OA Ob.

## Introduction

Osteoarthritis (OA) is the most common form of arthritis, and knee OA is amongst the most frequent type along with hip OA. OA is generally characterized by a gradual loss of cartilage in the articulation, sclerosis of the subchondral bone with the presence of osteophytes at the bone margin, and inflammation of the synovial membrane. As a final pathological manifestation, OA represents an imbalance between the loss of cartilage due to matrix degradation and the attempt to repair the matrix [1].

Abnormal osteoblasts (Ob) are involved in the onset and/or progression of OA [1, 2]. We previously reported that OA Ob demonstrate better cell proliferation [3] and an elevation in markers of differentiation such as alkaline phosphatase (ALPase), osteocalcin (OC) secretion, type 1 collagen production [4, 5] and growth factors such as transforming growth factor β1 (TGF-β1) [4, 6] compared to normal Ob. In clinical studies, the progression of joint cartilage degeneration is associated with increased bone stiffness as well as intensified remodeling of the subchondral bone [7]. OA patients have an increased bone mineral density, indicating that bone synthesis exceeds degradation [8]. However, femoral heads of patients with OA obtained at autopsy showed a low mineralization pattern compared to normal tissues [9, 10]. This may explain the apparent bone mineral density increase in OA as representing an increase in material density and not mineral density [11]. Indeed, the production of collagen type I and the ratio of collagen type I α1 to α2 chains, which is increased in OA tissue compared to normal, is the cause for an increase in undermineralized osteoid matrix [12]. This situation was observed in OA osteoblasts *in vitro* and linked to elevated levels of TGF-β1 [4].

Bone tissue remodeling and skeletal patterning involves a great number of signaling molecules and many signaling pathways are involved. More specifically, this involves the bone morphogenetic proteins (BMP), TGF-β1 and the Wnt protein family [13–15]. Indeed, our group reported that the canonical Wnt/β-catenin pathway is reduced in OA osteoblasts compared to normal osteoblasts [16]. The role of the canonical Wnt signaling pathway in OA has been shown in many studies [16–18], however, there is little research existing on the role and importance of the non-canonical (β-catenin independent) pathways in the onset and progress of OA. Since there is growing evidence that all Wnt pathways interact with each other as well as with other key signaling pathways [19–21], it is important to examine the role the non-canonical pathways could play in OA.

The two principal non-canonical Wnt pathways are the Wnt/PCP (planar cell polarity) [22] and the Wnt/PKC-Ca^2+^ (protein kinase C) [23]. These pathways have been described in different models. The Wnt/PCP uses Frizzled receptors to start a signaling cascade that uses small GTPases and eventually JUN-N-terminal kinase (JNK) to activate the final transcription factor c-JUN (AP1) [22, 24]. The Wnt/PCP and Wnt/β-catenin pathways are often antagonists [25] and we know that the latter is affected in OA [16, 17, 26]. Of note, we previously demonstrated that the stimulation of the canonical Wnt/β-catenin pathway by Wnt3a, which is the most potent stimulator of this pathway in bone tissue, is reduced in OA Ob [16]. Conversely, Wnt5a is the preferred Wnt/PCP activator ligand that binds to Frizzled 2 and could inhibit Wnt3a binding to that receptor, thus inhibiting Wnt/β-catenin signaling [27]. The Wnt/PKC-Ca^2+^ uses protein kinase C and Ca^2+^ release to activate the NFAT (nuclear factor of activated t-cells) transcription factor [28]. Both the Wnt/PCP and Wnt/PKC-Ca^2+^ pathways are activated via Wnt11 or Wnt5a [27, 29].

We previously showed that Wnt/β-catenin activity was reduced and that this was due to two possible mechanisms, an increase in the Wnt antagonist Dickkopf-2 (DKK2) [16] and a reduction of the non-Wnt agonist R-spondin 2 (Rspo2) in OA Ob [30]. It has indeed been shown that DKK2 is increased in response to an increase in TGF-β1 expression, and that this affects the phenotype of OA Ob. Also, Rspo2 could reduce the availability of the Frizzled receptor for other ligands besides Wnt3a by linking to the Leucine-rich G-coupled protein receptor (LGR) that acts as a co-receptor for the activation of the Wnt/β-catenin pathway. It would then be logical that a reduction in the Rspo2 activity would allow for a greater availability of Frizzled receptors thus allowing a greater activity of non-canonical Wnt pathways via Wnt5a activation. Of note, LGR4 and LGR5, two members of the LGR protein family [31, 32], are present in bone tissue [33, 34], whereas we have no information on their abundance and function in OA Ob.

This study was undertaken to evaluate and characterize the importance of the non-canonical Wnt/PCP and Wnt/PKC-Ca^2+^ signaling pathways in human OA osteoblasts.

## Material and methods

### Patients and clinical parameters

Tibial plateaus were obtained from OA patients undergoing total knee replacement surgery and prepared as previously described [5, 6]. Study patients (total of 42: 13 men and 29 women with a mean age ± SD of 68.88 ± 8.9 years) were classified as having OA according to the clinical criteria of the American College of Rheumatology. No patients had received medication that would interfere with bone metabolism, including corticosteroids, for 6 months before surgery. A total of 6 subchondral bone specimens from normal individuals (4 men and 2 women with a mean ± SD age of 61.33 ± 17.6 years) were collected at autopsy within 12 hours of death. These subjects had not taken any medication that could interfere with bone metabolism and had no reported bone metabolic disease or abnormal macroscopic changes of the cartilage upon visual inspection. All human samples were acquired following a signed agreement by the patients undergoing knee surgery and, for the specimens collected at autopsy, by the relatives of the deceased, in accordance with the ethics committee guidelines of the Centre de recherche du Centre Hospitalier de l’Université de Montréal (CRCHUM). The ethics review board of the CRCHUM approved the current study (BD.04.001-FIC).

### Preparation of primary subchondral bone cell cultures

The subchondral bone plate was isolated and the cell cultures were prepared as previously described [5]. At confluence, cells were passaged once at 25000 cells/cm^2^ and grown for 5 days in BGJb medium (Gibco) containing 10% fetal bovine serum (FBS). Confluent cells were then incubated in the presence or absence of 1,25-dihydroxyvitamin D_3_ (1,25[OH]_2_D_3_; 50 nM) for 48 hours for the determination of biomarkers. Supernatants were collected at the end of the incubation. Cells were prepared in alkaline phosphatase buffer (100 mM glycine, 1 mM MgCl, 1 mM ZnCl, 1% Triton X-100; pH 10.5) for protein determination and phenotype evaluation, in TRIzol^™^ for qRT-PCR experiments, or Laemmli buffer for Western blot analysis. Protein determination was performed by the bicinchoninic acid method. Wnt5a signaling activity was stimulated using recombinant human Wnt5a (rhWnt5a) protein at 100 ng/mL (R&D Systems) and TGF-β activity with recombinant human TGF-β1 (rhTGF-β1) at 10 ng/mL (R&D Systems). The expression of Wnt5a and TGF-β1 was inhibited in OA Ob by specific siRNA as previously described [5]. siWnt5a, siTGF-β1 and siScrambled (siSCR) preparations were Dharmacon SmartPOOL ONtarget products (a mix of 4 siRNA per target). For prolonged silencing treatments using siRNA in post-confluent osteoblasts, the treatments were repeated every 3 days of continuous culture.

### Phenotypic characterization of human subchondral Ob cell cultures

ALPase activity was determined by substrate hydrolysis using p-nitrophenylphosphate, and osteocalcin in cell supernatants using an EIA as previously described [5, 6]. Determinations were performed in duplicate for each individual cell samples prepared from normal individuals and OA patients.

### Evaluation of mineralization

Confluent cells were incubated in BGJb media containing 10% fetal bovine serum (FBS), 50 μg/ml ascorbic acid, 50 μg/ml β-glycerophosphate. This media was changed every two days until day 28 and cells were also stimulated with 10ng/ml BMP-2 every two days. BMP-2 is the most potent stimulator of mineralization and we previously demonstrated that it stimulated the mineralization of both normal and OA osteoblasts [4]. Mineralization of cell cultures was measured by quantification of alizarin red staining (ARS) with the procedure of Gregory et al [35]. Briefly, cells were fixed in 70% cold ethanol, incubated with 40 μM alizarin red at pH 4.1, washed and air-dried. Cells are then extracted with 10% acetic acid for 30 min, scraped from the Petri dishes, heated at 85°C for 10 min and then transferred on ice. An aliquot of the cell extract is incubated with 10% ammonium hydroxide and the color product read at 550 nm against a standard curve.

### Protein determination by western blotting

Cell extracts were prepared for WB as previously described [3, 30]. Rabbit anti-LGR5 (1:1000, Santa Cruz biotechnologies), Mouse anti-LGR4 (1:400 Santa Cruz biotechnologies), rabbit anti-*p*PKC (1:1000, Cell Signaling Technology), rabbit anti-*p*JNK (1:1000, Cell Signaling Technologies) and rabbit anti-human actin (1:10,000, Sigma-Aldrich) were used as primary antibodies, whereas goat anti-rabbit IgG and goat anti-mouse IgG (1:10,000, Upstate Biotechnology, NY) were used as secondary antibodies.

### qRT-PCR assays

RT reactions were primed with random hexamers with 1 μg of RNA followed by PCR amplification with the Rotor-Gene Q (Qiagen) as described (citation) using 20 pmol of specific PCR primers: TGF-β1, F:GCGTGCTAATGGTGGAAAC, R:GCTGAGGTATCGCCAGGAA; OC, F:ATG AGA GCC CTC ACA CTC, R:GAA AGC CGA TGT GGT CAG; GAPDH, F:CAG AAC ATC ATC CCT GCC TCT, R:GCT TGA CAA AGT GGT CGT TGA G; Wnt5a, F:TAA GCC CAG GAG TTG CTT TG, R:GCA GAG AGG CTG TGC TCC TA; LGR5, F:GGC TTT CTT GTC CTT CTC CT, R:CGT AGG TTT GCT TTC TCA GG; LGR4, F:GCA TCT TTT TCT GCC CTG TG, R:GAC AAC CAC CTT GGC TAC TG; LRP5, F:GCC ATC GAC TAT GAC CCA CT, R:CAG AAC AGT GTC CGG CTG TA; CBFA1, F:AGA TGA TGA CAC TGC CAC CTC TG, R:GGG ATG AAA TGC TTG GGA ACT GC; ALP, F:ACG TGG CTA AGA ATG TCA TC, R:CTG GTA GGC GAT GTC CTT A, added at a final concentration of 200 nM. The data were processed with the Rotorgene software, given as threshold cycle (Ct), Ct values converted to number of target gene molecules and values expressed as the ratio to GAPDH.

### NFAT, AP1 and TCF dual-reporter luciferase assays

Normal and OA Ob were plated in 24-well plates at a density of 1.5 × 10^5^ cells/well containing 10% FBS in BGJb media and left overnight. Plasmid mixtures containing either 2 μg NFAT, 2 μg AP-1, or 2 μg TOPflash luciferase construct (Qiagen and Upstate Biotechnology, Lake Placid, NY respectively) and 0.05 μg Renilla luciferase driven by the SV40 promoter (Promega) were transfected into cells overnight using the Transfectine (Bio-Rad) transfection Reagent according to the manufacturer's protocol. Media was changed and cells were left to recover from transfection for 6 h prior to incubation for 24 h with rh-Wnt5a (NFAT and AP-1 assays) or rh-Wnt3a (TOPflash), or a combination of both according to specific protocols. Cells were lyzed and luciferase activity evaluated using the dual luciferase assay kit (Promega). Values for NFAT, AP1 or TOPflash luciferase activities were normalized with Renilla activity for each individual preparation.

### Preparation of SaOS-2 cells

SaOS-2 cells are human osteoblast-like cells and were obtained from the American Type Culture Collection. SaOS-2 cells were grown in DMEM media containing 10% FBS. They were passaged once a week at a ratio of 1:6. At confluence, cells were fed with BGJb media (Sigma-Aldrich) containing 10% FBS, 50 μg/ml β-glycerophosphate and 50 μg/ml ascorbic acid to induce mineralization for 10 days [4]. SaOS-2 cells were also used for NFAT and AP1 luciferase assays as described above in response to rhWnt5a treatment for 24 hours.

### Statistical analysis

All experiments were performed either in duplicate or triplicate assays and the figure legends indicate the number of individual normal or OA Ob samples tested per conditions. Quantitative data are expressed as mean ± SEM. The data were analyzed by an ANOVA followed by multiple comparisons Tukey or Bonferroni-Dunn tests, and p values < 0.05 were considered statistically significant between subgroups.

## Results

### Production of Wnt5a, LGR4 and LGR5 in normal and OA osteoblasts

**Fig 1:**
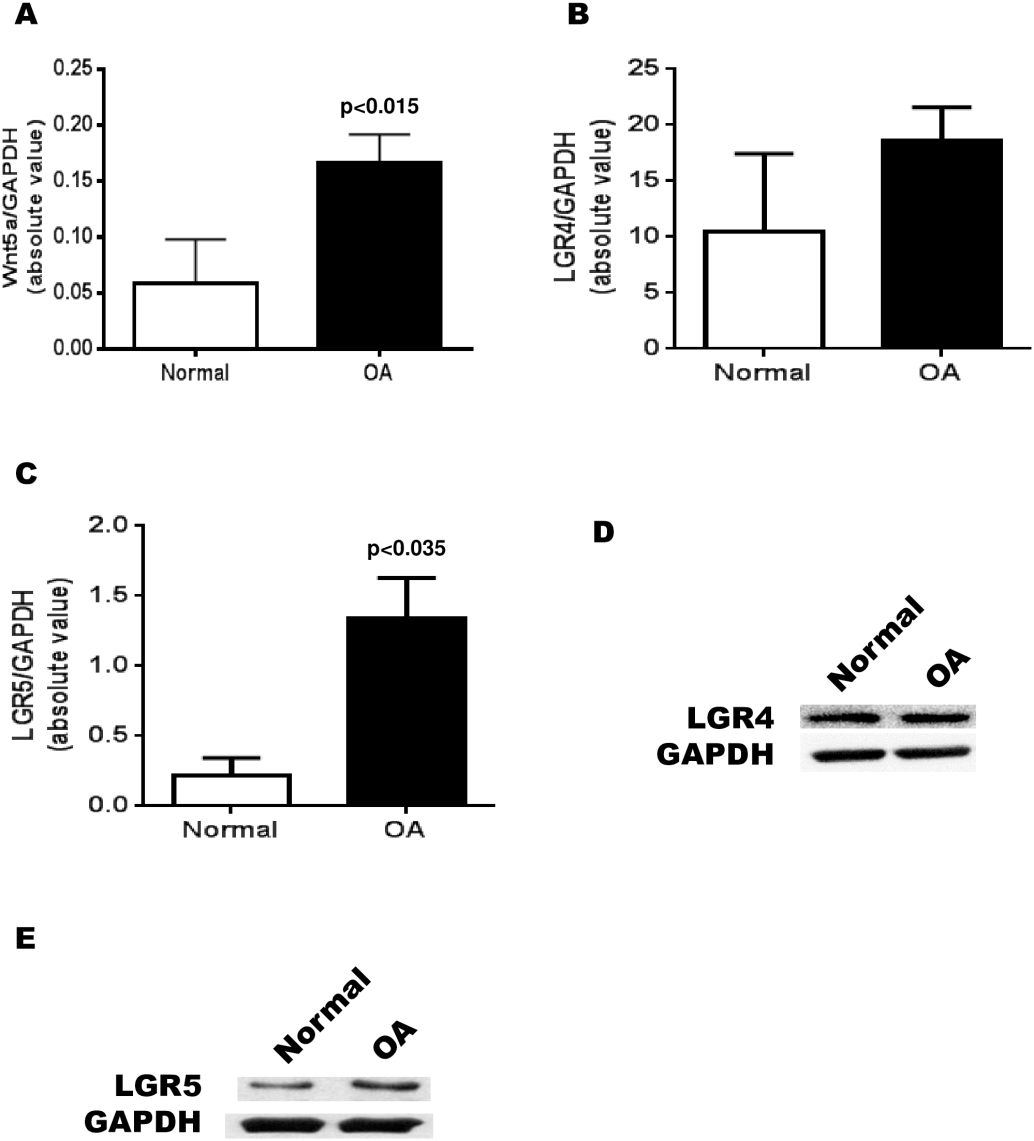
Expression of Wnt5a and LGR4 and LGR5 in normal and OA osteoblasts. Confluent normal and OA osteoblasts were prepared for RT-qPCR analysis. A) Wnt-5a expression in normal Ob (n = 4) and OA Ob (n = 16); B) LGR4 expression in normal Ob (n = 4) and OA Ob (n = 14); C) LGR5 expression in normal Ob (n = 4) and OA Ob (n = 14); D) Representative western blot of LGR4 production in normal and OA Ob; E) Representative western blot of LGR5 production in normal and OA Ob.

Previous studies have shown that there are a number of differences between OA and normal Ob [36, 37] in canonical Wnt pathway related genes, but there is little information as to the differences in non-canonical Wnt gene expression in human osteoblasts. We measured, using RT-qPCR, the difference in expression between normal and OA Ob of the principal non-canonical Wnt activator in Ob, Wnt5a. We were able to determine that there is ~5-fold increase in Wnt5a expression in OA Ob compared to normal Ob (Fig 1A) under basal conditions. Next, we measured the difference in expression between normal and OA Ob for the co-receptors LGR4 (Fig 1B) and LGR5 (Fig 1C). As observed herein, LGR4 is slightly increased although not significantly while the expression of LGR5 is increased ~5-fold in OA compared to normal Ob (p<0.01). Western blot analysis of LGR4 production by normal and OA Ob showed no significant differences (Fig 1D). In contract, LGR5 production by normal and OA Ob indicated an increase in LGR5 protein abundance in OA Ob compared to normal (Fig 1E). LGR5 abundance, relative to actin loading, was 0.49 ± 0.11 in normal Ob vs 0.86 ± 0.06 for OA Ob (mean relative value ± SEM of n = 3 separate preparations, p<0.05).

### Importance of the effect on Wnt-5a on OA Ob

**Fig 2:**
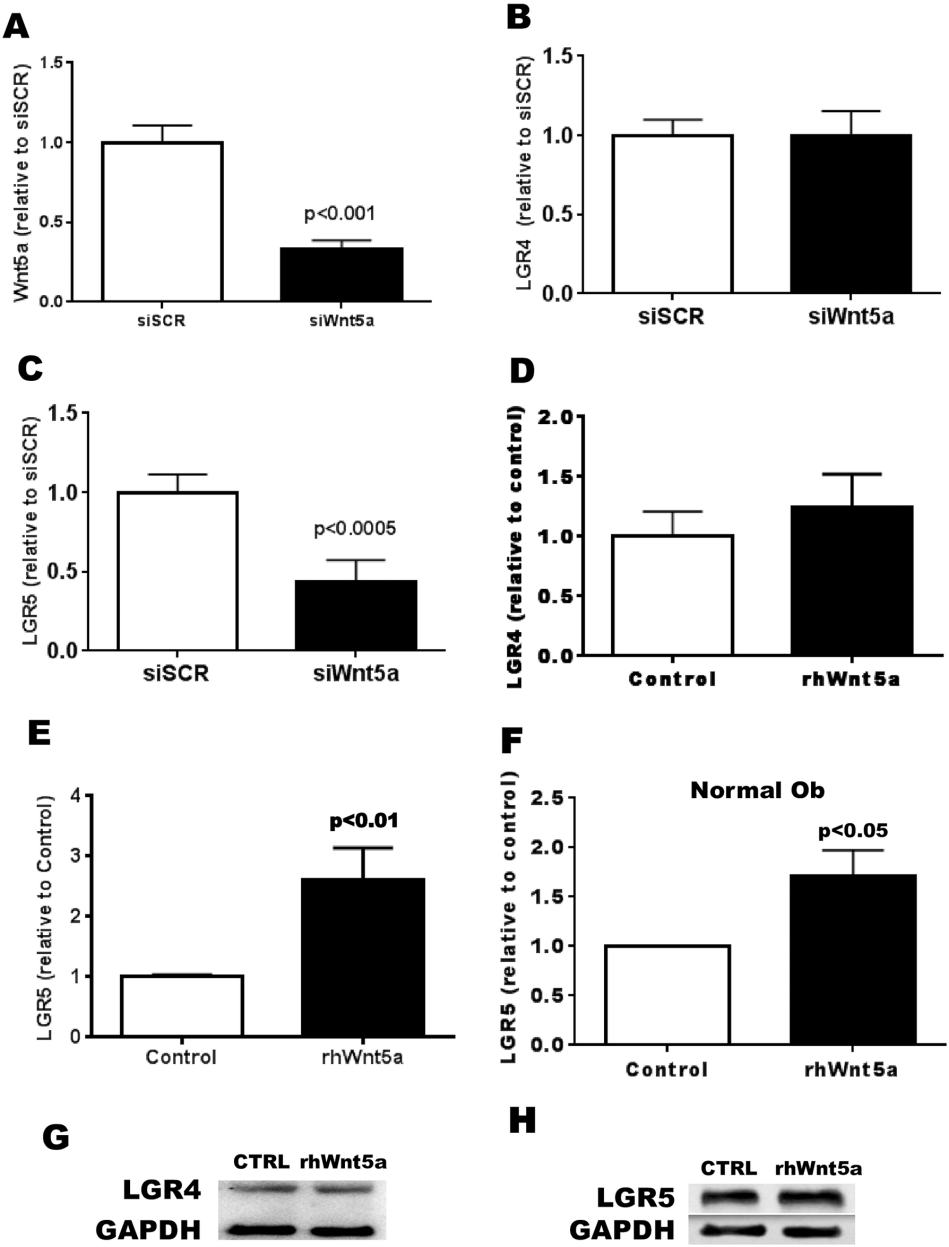
Regulation of the expression of LGR4 and LGR5 in OA osteoblasts. Confluent OA osteoblasts were treated with either recombinant human Wnt5a (100 ng/mL) or siWnt5a. LGR4 and LGR5 expression were measured by RT-qPCR. A. Effect of siWnt5a on Wnt5a gene expression (n = 6); B) LGR4 expression in response to siWant5a (n = 6); C) LGR5 expression in response to siWnt5a treatment (n = 6); D) LGR4 expression in response to rhWant5a (n = 7); E) LGR5 expression in response to rhWnt5a in OA Ob (n = 8); F) LGR5 expression in response to rhWnt5a in normal Ob (n = 3). G) Representative western blot of LGR4 production in OA Ob stimulated by rhWnt5a for 48 hours; H) Representative western blot of LGR5 production in OA Ob stimulated by rhWnt5a for 48 hours.

Since Wnt5a is a known activator of the non-canonical pathways and could regulate its own receptors, we then wanted to test if the augmented expression of LGR5 could be linked to the elevated Wnt5a expression that we measured. Indeed, following our observation that the production of Wnt5a in OA Ob cells is increased and that an increase in its concentration can increase known markers of non-canonical Wnt signaling pathways, we next wanted to measure the effect of a reduction of Wnt5a expression in OA Ob. To test this, we first used a small interfering RNA (siRNA) approach to reduce the expression of Wnt5a (siWnt5a). After 48 hours of siWnt5a treatment, we were able to reduce the expression of Wnt5a in primary OA Ob cells by an average of 67.3 +/- 5.8% (p<0.001) (Fig 2A), which brings the expression level relative to GAPDH closer to the range of expression in normal Ob. Second, after reducing the expression of Wnt5a using this siRNA technique, we measured its effect on LGR gene expression. Using RT-qPCR, we were able to measure an average reduction of almost 50% of the expression of LGR5 following Wnt5a inhibition (Fig 2C). In contrast, we were unable to detect a significant decrease in the expression of LGR4 after the siWnt5a treatment (Fig 2B). Third, following the treatment of OA Ob cells with rhWnt-5a (100 ng/mL, 24h incubation), we detected an increase in the relative LGR5 expression of about 2.5-fold (Fig 2E). In contrast, the expression of LGR4 was not stimulated by the same treatment (Fig 2D). Stimulating OA Ob with rhWnt5a did not increase the abundance of LGR4 proteins as detected by Western blot analysis (Fig 2G) whereas the same treatment induced a small but significant increase of LGR5 abundance (Fig 2H). Indeed, rhWnt5a induced a 24.5% increase in LGR5 abundance in OA Ob (p<0.05). In addition, a 24 hours treatment with rhWnt5a induced a 39% increase (p<0.05) in LGR5 mRNA abundance in normal Ob (Fig 2F).

**Fig 3:**
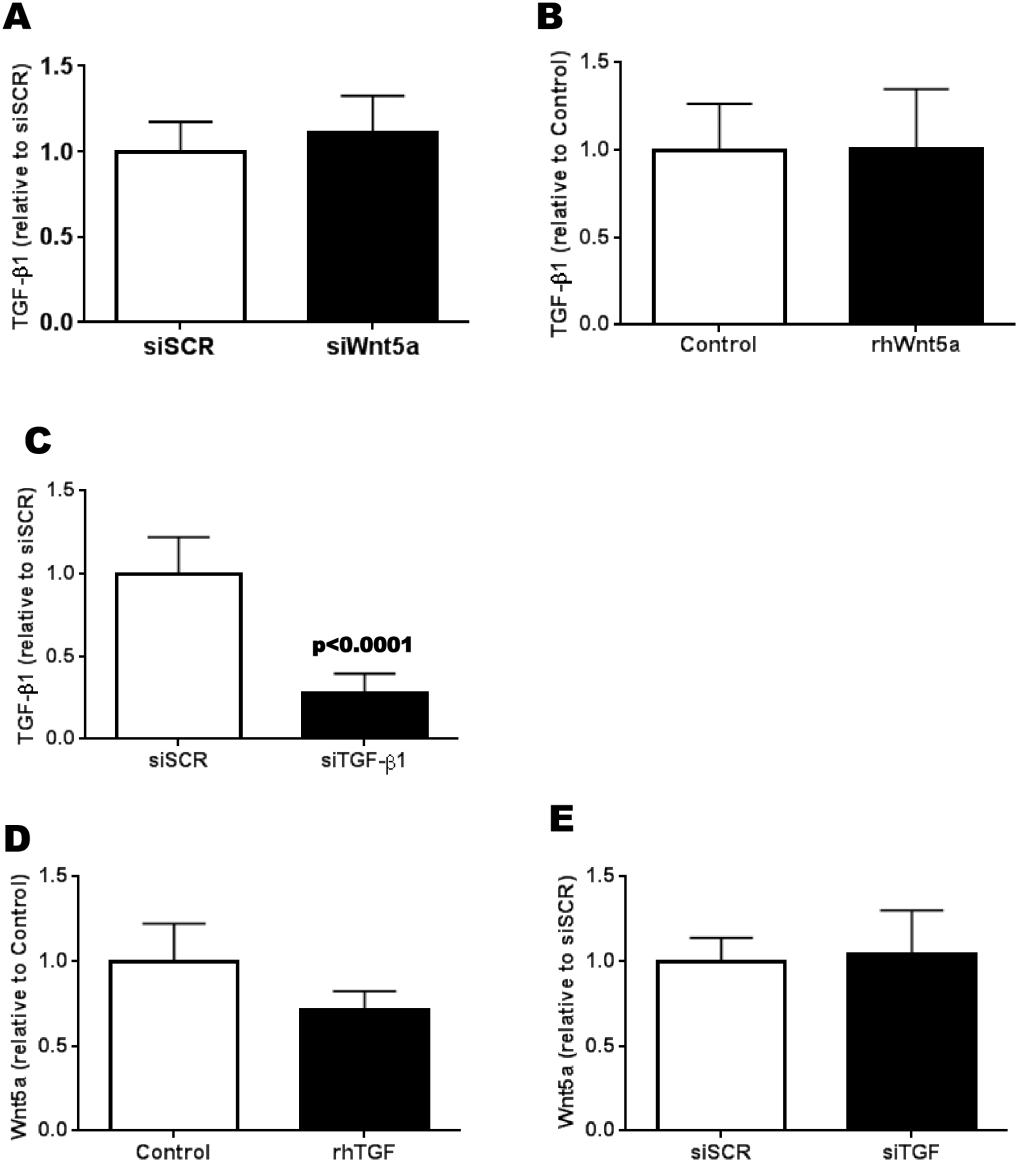
Reciprocal regulation of Wnt5a and TGF-β1 expression in OA osteoblasts. Confluent OA osteoblasts were treated with either rhWnt5a or siWnt5a in order to measure the expression of TGF-β1. Conversely, OA osteoblasts were treated with either rhTGF-β1 or siTGF-β1 prior to the measure of Wnt5a expression. Gene expression was measured by RT-qPCR. A) Expression of TGF-β1 in response to siWnt5a treatement (n = 4); B) Expression of TGF-β1 in response to rhWnt5a (n = 3); C) Expression of TGF-β1 in response to siTGF-β1 treatment (n = 6); D) Expression of Wnt5a in response to rhTGF-β1 (n = 3); E) Expression of Wnt5a in response to siTGF-β1 (n = 4).

Since we previously demonstrated that endogenous levels of TGF-β1 in OA Ob are responsible for the alterations of the Wnt/β-catenin signaling pathway [4], here we also tested if TGF-β1 could be linked to Wnt5a expression. First, we tested if Wnt5a levels could affect the expression of TGF-β1. The addition of either siWnt5a or rhWnt5a had no effect on the expression of TGF-β1 in OA Ob (Fig 3A and 3B). We next verified that siTGF-β1 could reduce the expression of TGF-β1 which was the case with an average of 72 +/- 4.8% reduction (Fig 3C). Last, the addition (Fig 3D) of rhTGF-β1 or its reduction by siRNA (Fig 3E) showed no effect on Wnt5a expression.

### Effect of the reduction of Wnt5a on gene expression over time

**Fig 4:**
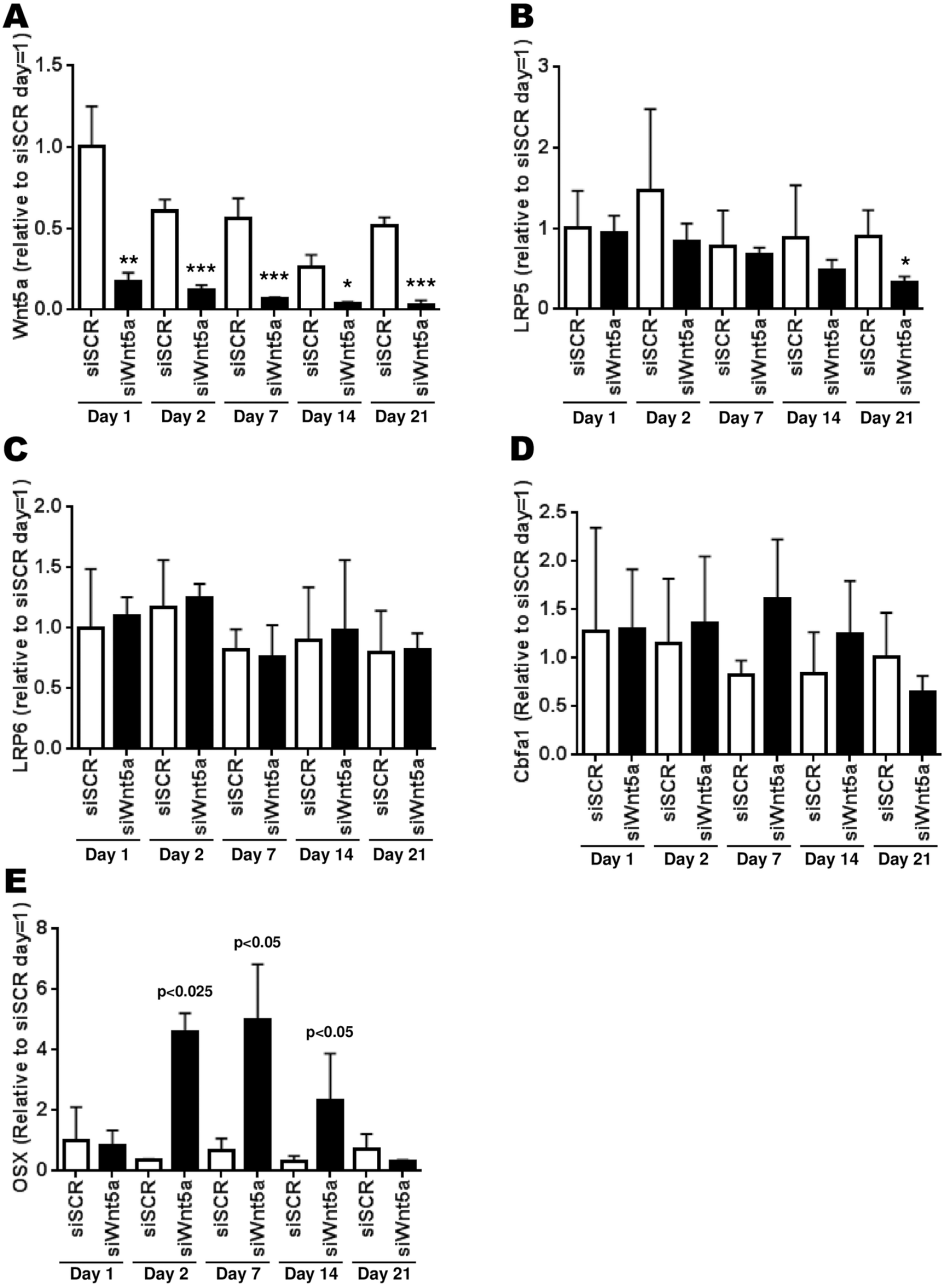
Time-dependent effect of siWnt5a treatment on gene expression of targeted genes. Confluent OA osteoblasts were treated with siWnt5a at time = 0 and every three days until 21 days of treatment. Osteoblasts were lyzed at the indicated time points and prepared for RT-qPCR. A) Expression of Wnt5a during the 21 days of osteoblast differentiation; B) Regulation of LRP5 expression; C) Regulation of LRP6 expression; D) Regulation of CBFA1/Runx2 expression; E) Regulation of Osterix expression. The results are the mean +/- SEM of n = 7 preparations. Statistical significance of siWnt5a treatments vs siSCR are indicated directly on the figure: * p<0.05, ** p<0.01 and *** p<0.005.

After having tested for genes that were affected after an acute treatment of 24h to 48h, we wanted to measure the expression of key genes involved in osteogenesis over a period of time up to 21 days post-confluence using a sustained siWnt5a treatment and if this could modulate these genes. First, we measured whether the expression of Wnt5a would change over 21 days after confluence in OA Ob. Whereas a slight decrease in Wnt5a expression was noted over time under basal condition, no significant changes was achieved through this timeline (Fig 4A). However, siWnt5a treatments resulted in a sustained inhibition of Wnt5a expression at all time- points tested post-confluence (Fig 4A). Second, the expression of LRP5, LRP6, CBFA1/Runx2 and Osterix (OSX) under basal conditions remained stable over the 21 days post-confluence (Fig 4B, 4C and 4D). However, the treatment with siWnt5a reduced significantly the expression of LRP5 up to less than half its control level only after 21 days post-confluence (Fig 4B). However, the same treatment with siWnt5a was without effect for LRP6 (Fig 4C) and marginal for CBFA1/Runx2 (Fig 4D). Although all the differences are not significant for CBFA1/Runx2, it is possible to observe a general tendency for an increase in its expression. Indeed, the siWnt5a treatment increases the Runx2 expression for the first 7 days up to ~1,5 times the control level, then it has the opposite effect for longer incubation time-points (Fig 4D). The expression of OSX also seems to be modulated in the same manner as for CBFA1/Runx2. However, the variations are quite significant at days 2, 7 and 14 post-confluence, increasing by up to 5-fold the expression of OSX and then decreasing below the control level (Fig 4E).

### Phenotypic characterization of osteoblasts/in vitro mineralization and phenotypic markers following Wnt5a reduction

**Fig 5:**
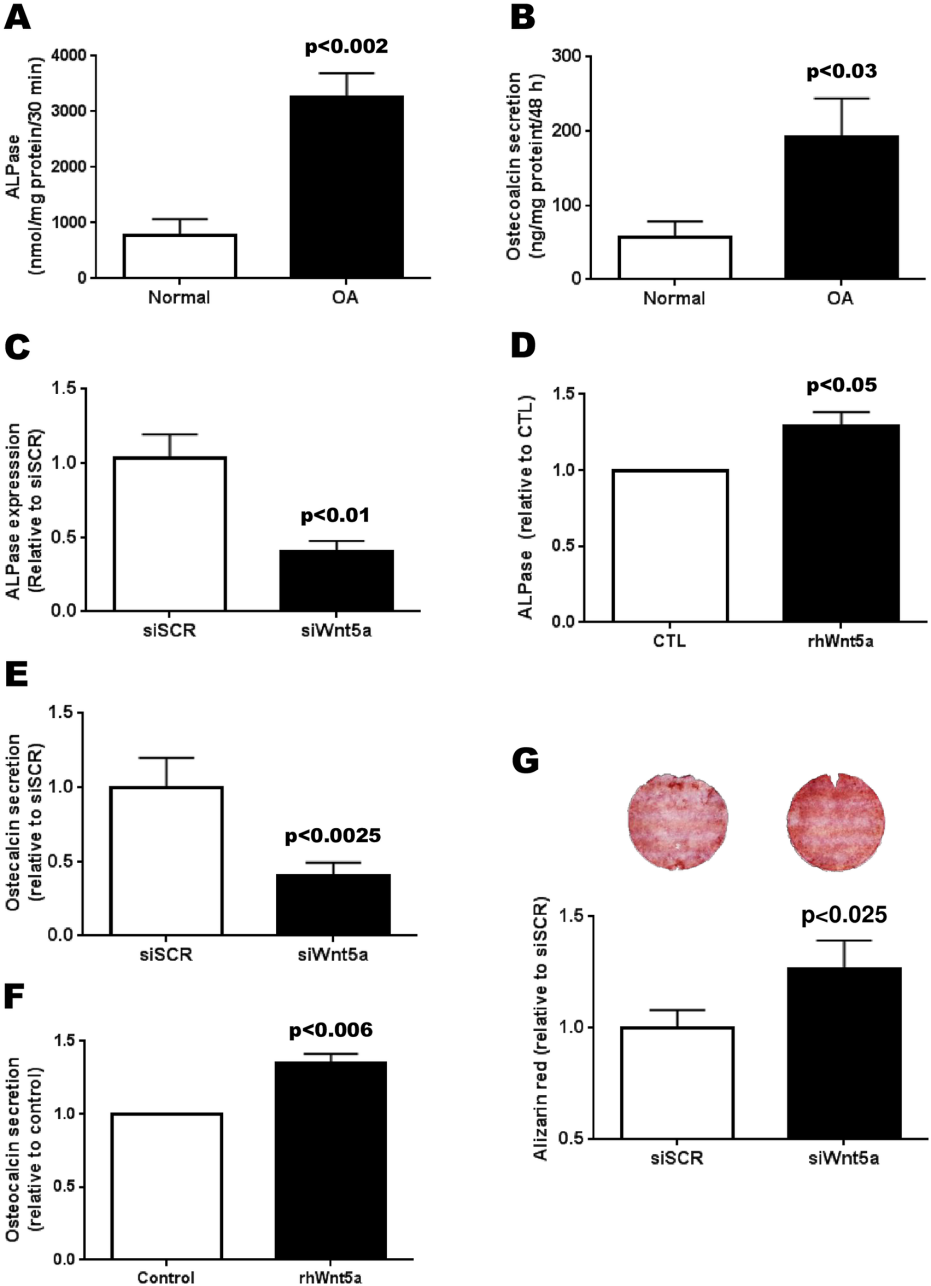
Effect of Wnt5a regulation on the phenotype of osteoblasts. Confluent normal (n = 6) and OA osteoblasts (n = 42) were stimulated with 50 nM 1,25(OH)_2_D_3_ prior to the determination of alkaline phosphatase activity (A) and osteocalcin secretion (B). OA osteoblasts were also treated with either siWnt5a of rhWnt5a and the activity of alkaline phosphatase or osteocalcin secretion was measured after 1,25(OH)_2_D_3_ stimulation. C) Effect of siWnt5a treatment on the expression of TNAP (n = 5); D) Effect of rhWnt5a on ALPase activity levels in normal Ob (n = 3); E) Effect of siWnt5a treatment on osteocalcin secretion in OA Ob (n = 8 preparations); F) Effect of rhWnt5a on osteocalcin secretion in normal Ob (n = 3). G) Effect of siWnt5a treatment for 28 days on mineralization levels of OA osteoblasts as measured with Alizarin red staining (n = 5 preparations). Top: representative ARS staining following siSCR or siWnt5a treatment.

As was previously reported, ALPase activity (Fig 5A) and OC secretion (Fig 5B) were high in all OA Ob compared to normal [4, 5]. We therefore tested whether the increase in Wnt5a detected in OA Ob could be linked with these parameters. We first measured the effect of the siWnt5a treatment on the expression of alkaline phosphatase (TNAP) and ostecalcin. There was a decrease of about 50% in the expression of the TNAP in response to siWnt5a (Fig 5C). Conversely, the effect of a 100 ng/mL treatment with rhWnt5a stimulated in the activity of alkaline phosphatase (Fig 5D), which is associated with OA. Next, we tested the effect of siWnt5a on osteocalcin expression and we noted no significant effect on this activity (not illustrated). Conversely, siWnt5a treatment decreased OC secretion by 40% (Fig 5E). In addition, the treatment with rhWnt5a stimulated osteocalcin secretion in OA Ob (Fig 5F). Finally, it has been reported that mineralization levels in OA Ob are lower than in normal Ob [4]. We then questioned if abnormally high Wnt5a levels could be linked with this reduction in mineralization in OA Ob. Inhibiting Wnt5a expression by siRNA during at least 28 days showed a significant increase in mineralization of ~30% (Fig 5G).

### Effect on non-canonical signalisation in OA Ob

**Fig 6:**
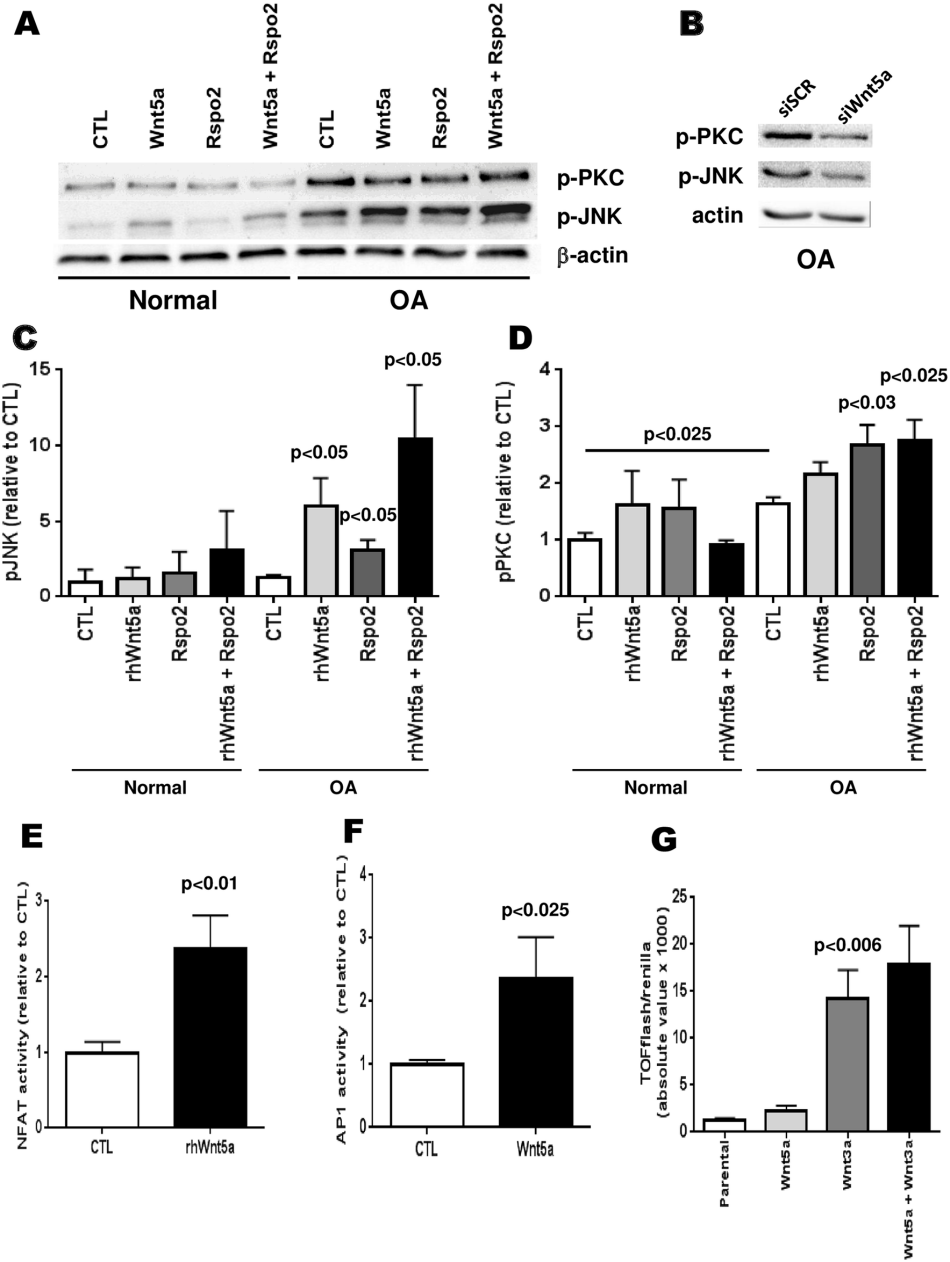
Effect of Wnt5a and Rspo-2 treatments on non-canonical Wnt pathway signaling in normal and OA osteoblasts. Confluent normal and OA osteoblasts were treated with rhWnt5a, rhRspo2 or both for 15 minutes. Proteins were prepared from cells for Western blot analysis. A) Representative Western blot analysis of normal and OA osteoblasts; B) Representative Western blot analysis for phospho-PKC and phospho-JNK of OA osteoblasts treated with siWnt5a or a siSCR (control); C) Quantification of the phosphorylation of JNK (*p*-JNK) in normal and OA osteoblasts relative to actin loading (OA: n = 5, normal: n = 3); D) Quantification of the phosphorylation of PKC (*p*-PKC) in normal and OA osteoblasts relative to actin loading (OA: n = 4 and N: n = 3); E) NFAT activity level measured with dual-luciferase reporter assay following rhWnt5a treatment in OA osteoblasts (n = 8); F) AP-1 activity level measured with dual-luciferase reporter assay following rhWnt-5a treatment in OA osteoblasts (n = 3); G) TOPflash activity level in response to Wnt3a, Wnt5a or both in OA osteoblasts (n = 4).

To find what could cause the effects that were measured previously, we then verified if the Wnt non-canonical signalisation pathways were affected in OA Ob linked with the abnormal expression of Wnt5a. To ascertain this, we tested if Wnt5a would increase the activity of the Wnt/PCP and Wnt/PKC pathways in Ob cells, and if Rspo2, which was previously described to have an impact on the Wnt canonical signaling pathway in OA [30], would act as a co-activator of these pathways. First, using western blot analysis, the phosphorylation of JNK was evaluated for the Wnt/PCP pathway and the phosphorylation of PKC for the Wnt/PKC pathway for normal and OA osteoblasts (Fig 6A). Since basal levels of phospho-PKC and phospho-JNK were increased in OA osteoblasts compared to normal osteoblasts (Fig 6A), we tested if this was linked with elevated Wnt5a levels in these cells. Inhibiting Wnt5a expression by siRNA reduced both phospho-PKC (37.1 ± 11.1%, p<0.025 vs siSCR) and phospho-JNK (20.8 ± 3.5%, p<0.025 vs siSCR) in OA osteoblasts (Fig 6B). We were also able to detect that rhWnt5a increases the phosphorylation of JNK and that Rspo2 has only a slight effect by itself but when used in combination with Wnt5a, this boosts its effect (Fig 6A and 6C). The effect of Wnt5a on the phosphorylation of PKC was much smaller than for JNK yet Rspo2 seems to have a greater relative effect on it (Fig 6A and 6D).

We then tested if the observed effect of Wnt5a would be confirmed by the activation of the NFAT transcription factor, which should normally be involved in the activation of the Wnt/PKC pathway. Using a dual-luciferase NFAT reporter assay, we were able to detect a ~2.4-fold increase in the activation of NFAT (Fig 6E), showing that the increase in activity of this pathway goes all the way to the transcription factor. We also evaluated the activity of the other pathway (Wnt/PCP) via the activation of the AP-1 transcription factor and we were able to increase the AP-1 activity also about 2.4-fold following a treatment with rhWnt5a (Fig 6F). Last, we tested whether the presence of increased Wnt5a levels could alter the Wnt3a-dependent canonical Wnt/β-catenin signaling in OA Ob. As illustrated by Fig 6G, Wnt3a was able to stimulate TOPflash luciferase activity in OA Ob about 10-fold whereas Wnt5a alone could not. In addition, the presence of Wnt5a did not influence the response to Wnt3a under these conditions in these cells. Moreover, using SaOS-2 cells, we were also able to demonstrate that rhWnt5a stimulate AP-1 (S1 Fig) and NFAT (S2 Fig) activities. In addition, rhWnt5a reduced SaOS-2 cells mineralization (S3 Fig)

## Discussion

The Wnt signaling pathway is important for normal skeletal tissue homeostasis and function. Bone, and in particular the subchondral bone tissue, is abnormal in OA patients [9, 11, 38], and we previously showed that OA subchondral osteoblasts have altered functions compared to normal that can explain, in part, these differences [4, 5, 39]. The canonical Wnt/β-catenin signalisation is important for the function of subchondral osteoblasts, but there is no of a role for the non-canonical Wnt pathways in these cells or for OA. Indeed we previously reported a reduction in the Wnt agonist R-spondin 2 [30], a stimulation of the Wnt antagonists DKK2 [16] and SOST [40], along with increases in TGF-β1 [16, 40] which can cause an imbalance in the canonical Wnt/β-catenin signaling pathway. It is believed that all Wnt pathways are somewhat linked [19]. If so, this inhibition of the Wnt/β-catenin pathway in OA osteoblasts could then cause an imbalance of the non-canonical Wnt pathways in these cells. We show in this study that the non-canonical Wnt/PKC and Wnt/PCP pathways are indeed affected in OA osteoblasts, and this can be linked to the abnormal expression of the non-canonical Wnt agonist Wnt5a.

We also found that the expression and production of the non-canonical Wnt agonist Wnt5a was actually increased in OA Ob compared to normal Ob along with the Wnt co-receptor LGR5. In fact this increase in Wnt5a expression stimulates the LGR5 expression and production in OA osteoblasts, which could then be a potential link to the stimulation of the non-canonical pathways. It is noteworthy that the increased Wnt5a expression observed in OA Ob lead to an up-regulation of LGR5, but not LGR4, both at the mRNA level and protein level. In addition, the observation that rhWnt5a can stimulate LGR5 expression in both normal and OA Ob further indicates that Wnt5a can regulate this Wnt/non-canonical pathway. Indeed, as previously observed by Glinka *et al*. [41], both LGR4 and LGR5 can mediate Wnt-PCP signaling, hence if Wnt5a is able to influence LGR4 or LGR5, it could affect this non-canonical pathway in this manner. This concurs with our previous observation that R-spondin 2 is reduced in OA Ob [30]; Rspo-2 binds to a co-receptor containing LGR4, 5 or 6 thus reducing its availability [19]. The low R-spondin 2 level previously observed in OA Ob would allow for a greater availability of LGR5, which could then be used to control the non-canonical Wnt pathways activation.

Knowing that TGF-β1 is a key player in OA pathology [42–44] and is involved in the abnormal phenotype of OA Ob [16] relative to the Wnt β-catenin pathway, we also evaluated if it could be linked to the observed imbalance of the non-canonical Wnt pathways. However, our data indicate that the expression of TGF-β1 is independent of Wnt5a whereas the expression of Wnt5a is independent of TGF-β1. Hence, at present we do not exactly know what drives the spontaneous expression of Wnt5a in OA Ob under basal conditions such as those tested herein. However, our data also suggests that Wnt5a is also a key pathway altered in OA pathology.

The low-density lipoprotein receptor-related protein (LRP) 5 and 6 are known to be involved in the Wnt/β-catenin pathway [19, 45]. These receptors are linked with Frizzled receptors and act as co-receptors for Wnt signaling. To further investigate the interplay between the canonical and non-canonical pathways, we validated if there was indeed an effect of Wnt5a on a number of genes involved in Wnt signaling and osteogenesis. First, the inhibition of elevated Wnt5a levels in OA osteoblasts caused a clear drop in relative LRP5 expression 21 days post-confluence, which could suggest a reduction in the Wnt canonical pathway activity in these cells as they become more differentiated. However, this effect seems to be limited to LRP5, as LRP6 expression was unaffected by the same treatment which may prevent any significant effect on the Wnt canonical signaling pathway. We also verified the effect of the inhibition of Wnt5a over time on 2 key players in osteoblastic differentiation, CBFA1/Runx2 [46] and Osterix [47]. Although the increase in the expression of CBFA1/Runx2 did not reach significance in response to the inhibition of Wnt5a, the same treatment lead to a significant yet transient increase of the expression of Osterix within the first two weeks, potentially helping the osteoblasts to differentiate.

However, while this inhibition of Wnt5a was sufficient to transiently induce Osterix expression, and potentially promote Ob differentiation, it also promoted Ob mineralization. As there is little to no information on the effect of Wnt5a on bone formation and mineralization in human, it is interesting to note that we were able to slightly increase the mineralization of OA osteoblasts while reducing endogenous Wnt5a expression. Treating human osteoblast-like osteosarcoma SaOS-2 cells with rhWnt5a for 10 days reduced the mineralization capacity of these cells (S3 Fig), suggesting that Wnt5a has a direct effect on the mineralization potential of osteoblasts. Subchondral bone plate thickening and bone sclerosis are now consistent clinical findings in OA [10], but we now know that this is not linked with an increase in bone formation *per se*. This appears to be due to an increase in the formation of the bone type 1 collagen extracellular matrix with an imbalance in α1 to α2 chains leading to a reduced mineralization [4, 12]. Therefore, this overexpression of Wnt5a in OA osteoblasts could be in part responsible for this abnormal mineralization, along with other factors like SOST [40]. The expression and secretion of osteocalcin have been shown to be increased in OA Ob [4], and we have been able to measure a reduction in OC secretion following the inhibition of Wnt5a. Interestingly, the expression levels of osteocalcin were not affected by Wnt5a, indicating that Wnt5a directly affects OC secretion but not its transcription. OC secretion is regulated by a number of mechanisms involving 1,25(OH)_2_D_3_, PTH, IGF-1, TGF-β1 and leptin [48, 49]. Alkaline phosphatase activity is commonly elevated in OA Ob and this also contributes to the disturbed mineralization process that is observed. This makes our finding that Wnt5a can influence the increase alkaline phosphatase activity as well as its expression levels interesting.

Although the non-canonical Wnt pathways have been characterized in different cell lines [19, 50], there is little to no evidence that the same factors are involved in human bone tissue and in OA Ob. To evaluate the role of each pathway, we used a dual approach of measuring the phosphorylated forms of PKC and JNK and we determined the activation of their transcription factors, NFAT and AP1 respectively in response to Wnt5a stimulation. First, our data suggested that basal levels of phospho-PKC and phospho-JNK were increased in OA Ob compared to normal and indeed inhibiting Wnt5a by siRNA technique in OA Ob reduced their protein production. Our data also clearly demonstrated a robust activation of the Wnt/PCP pathway in response to Wnt5a in OA Ob, consistent with the level of phosphorylation of JNK in response to Wnt5a. This also resulted in a robust 2.4-fold increased activity of the AP1 luciferase transcriptional activity. In contrast, for the Wnt/PKC pathway, the response to Wnt5a was weaker in OA Ob at the protein level, possibly because the activity of this pathway is already high in OA Ob compared to normal Ob. Indeed, inhibiting Wnt5a by siRNA technology showed a more robust decrease in phospho-PKC in OA osteoblasts compared to phospho-JNK which would suggest that further stimulation of phospho-PKC would be difficult to observe in OA Ob in response to exogenous Wnt5a. However, we were able to detect a 2.4-fold increase of the Wnt/PKC pathway at the transcriptional activity level as reported by the dual NFAT/Renilla reporter assay. Since the phosphorylation of PKC was rather small in OA Ob in response to Wnt5a stimulation, this suggests that the NFAT activity could also be stimulated at another level than only at the *p*-PKC level in OA Ob, and this would require further investigation.

In normal cells, previous studies indicated an increase ranging from 35 to 300% of NFAT and AP1 signaling in response to Wnt5a [51–54]. We tested the response to rhWnt5a in SaOS-2 cells and observed that NFAT signaling was stimulated by about 19.5 ± 2.5% whereas the AP1 signaling was stimulated by about 13± 2.5% (S1 and S2 Figs). Also, both with *p*-JNK and *p*-PKC, the addition of Rspo-2 in conjunction with Wnt5a, but not alone, was able to boost the effect of Wnt5a supporting the idea that Rspo-2 is crucial to further stimulate the non-canonical pathways. Indeed, Rspo-2 on its own cannot stimulate the Wnt non-canonical pathways yet it promotes the effect of non-canonical Wnts on these pathways via LGRs [55–57]. Further, we were able to demonstrate that Wnt5a alone cannot stimulate the canonical Wnt/β-catenin signaling pathway in OA Ob whereas it also does not inhibit nor increase the stimulation observed in response to Wnt3a alone. This is interesting since the response to Wnt5a stimulation is either stimulatory or inhibitory for the Wnt/β-catenin pathway and cell and context-dependent [50, 58, 59] in different cell types. Herein, we clearly showed that Wnt5a alone was without significant effect on the canonical Wnt/β-catenin pathway in OA Ob whereas it had a clear effect on the non-canonical NFAT and AP1 signaling pathways.

## Conclusion

In conclusion, the findings of this study indicate that Wnt5a plays an important role in both the non-canonical Wnt/PCP and Wnt/PKC pathways in OA osteoblasts. The elevated levels of Wnt5a altered non-canonical Wnt signaling pathways and affect both the phenotype and the mineralization process of these cells. This demonstrates, in addition to the previously observed alteration of the canonical Wnt pathway in OA Ob, that the three major Wnt signaling pathways are altered in OA.

## Supporting information

S1 Fig Effect of rhWnt5a treatments on AP-1 luciferase activity. AP-1 activity levels were measured with dual-luciferase reporter assay following rhWnt5a treatment in SaOS-2 cells (n = 3).
(PDF)

S2 Fig Effect of rhWnt5a treatments on NFAT luciferase activity. NFAT activity levels were measured with dual-luciferase reporter assay following rhWnt5a treatment in SaOS-2 cells (n = 4).
(PDF)

S3 Fig Effect of rhWnt5a on SaOS-2 cells mineralization. Representative mineralization levels using ARS staining of SaOS-2 cells following rhWnt5a treatments for 10 days.
(PDF)

S1 File Supporting absolute data for LGR4 expression in normal and OA osteoblasts. (PZFX)

S2 File Supporting absolute data for LGR5 expression in normal and OA osteoblasts. (PZFX)

S3 File Supporting absolute data for effect of rhTGF-β on Wnt5a expression in OA osteoblasts. (PZFX)

S4 File Supporting absolute data for effect of rhWnt5a on TGF-β expression in OA osteoblasts. (PZFX)

S5 File Supporting absolute data for effect of siTGF-β on Wnt5a expression in OA osteoblasts. (PZFX)

S6 File Supporting absolute data for effect of siWnt5a on alizarin red staining in OA osteoblasts. (PZFX)

S7 File Supporting absolute data for effect of siWnt5a on LGR4 expression in OA osteoblasts. (PZFX)

S8 File Supporting absolute data for effect of siWnt5a on LGR5 expression in OA osteoblasts. (PZFX)

S9 File Supporting absolute data for effect of siWnt5a on TGF-β expression in OA osteoblasts. (PZFX)

S10 File Supporting absolute data for effect of rhWnt5a on AP1 luciferase activity in OA osteoblasts. (PZFX)

S11 File Supporting absolute data for effect of rhWnt5a on NFAT luciferase activity in OA osteoblasts. (PZFX)

S12 File Supporting absolute data for Wnt5a expression in normal and OA osteoblasts. (PZF)
