# Supplementary figures and images for: Alteration of Wnt5a expression and of the non-canonical Wnt/PCP and Wnt/PKC-Ca2+ pathways in human osteoarthritis osteoblasts
Source: PLoS One. 2017 Aug 4;12(8):e0180711. doi: 10.1371/journal.pone.0180711 (PMC5544184; doi:10.1371/journal.pone.0180711)

**S1 Fig.**

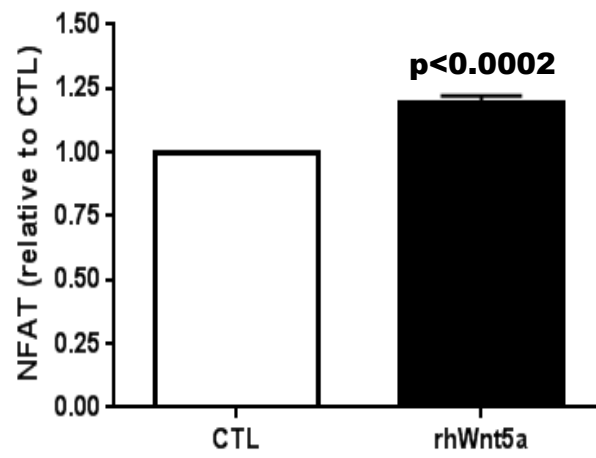

Supplement: S1 Fig — AP-1 activity levels were measured with dual-luciferase reporter assay following rhWnt5a treatment in SaOS-2 cells (n = 3). (PDF) [file pone.0180711.s001.pdf]

**S2 Fig.**

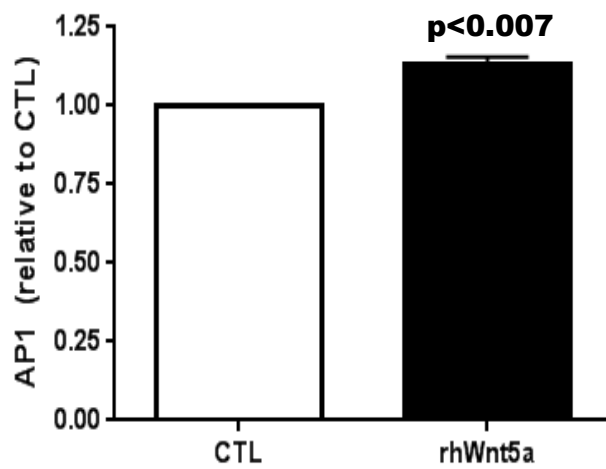

Supplement: S2 Fig — NFAT activity levels were measured with dual-luciferase reporter assay following rhWnt5a treatment in SaOS-2 cells (n = 4). (PDF) [file pone.0180711.s002.pdf]

**S3 Fig.**

**SaOS-2 cells**

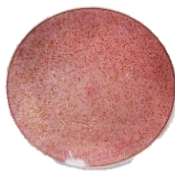

**CTL**

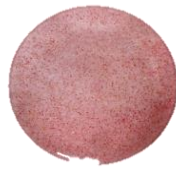

**rhWnt5a**

Supplement: S3 Fig — Representative mineralization levels using ARS staining of SaOS-2 cells following rhWnt5a treatments for 10 days. (PDF) [file pone.0180711.s003.pdf]
